# Predicting the Clean Movement Technique in Crossfit^®^ Athletes Using an Optimal Upper-Limb Range of Motion: A Prospective Cohort Study

**DOI:** 10.3390/ijerph191912985

**Published:** 2022-10-10

**Authors:** Antonio Cejudo

**Affiliations:** 1 Department of Physical Activity and Sport, Faculty of Sport Sciences, CEIR Campus Mare Nostrum (CMN), University of Murcia, 30720 Murcia, Spain; antonio.cejudo@um.es; Tel.: +34-868-888-430; 2 Locomotor System and Sport Research Group (E0B5-07), University of Murcia, 30720 Murcia, Spain

**Keywords:** weightlifting, high-intensity interval training, prevention, flexibility, flexibility assess

## Abstract

Background: The aim of this study was to determine the optimal upper-limb range of motion (ROM) profile for the catch phase of the clean movement (CPCM) and to identify the key ROMs for performing the CPCM in CrossFit^®^ athletes. Methods: A prospective cohort study of twenty CrossFit^®^ athletes aged 20–36 years was conducted. Data were collected regarding age, anthropometrics, CrossFit^®^ training experience and upper-limb ROM. The ROM was measured using the ROM-SPORT method. After 7 months, athletes performed a clean movement with a load of 80% one repetition maximum. A Bayesian Student’s t-analysis, binary logistic regression analysis and Receiver Operating Characteristic analysis were performed. Results: The optimal upper-limb ROM profile that predicted correct CPCM performance was 78° in shoulder extension, 173° in shoulder flexion, 107° in shoulder external rotation, 89° in shoulder internal rotation, 153° in elbow flexion, 99° in elbow pronation and 92° in wrist extension (area under the curve ≥ 651; positive predictive value ≥ 80%). Shoulder external rotation, elbow pronation and wrist extension were found to be the most important ROMs for the efficient and safe performance of CPCM (area under the curve ≥ 854; positive predictive value ≥ 85.7%). Conclusion: The upper-limb ROM profile is associated with proper clean performance. Further studies are warranted to determine whether improving flexibility on upper-limb ROM may improve proper clean movement performance.

## 1. Introduction

CrossFit^®^ is a movement with over 14,000 gyms and over 5 million athletes around the world [1]. This sport is a branded fitness training program that involves constantly varied functional movements performed at high intensity [2]. The training programme includes bodyweight and resistance exercises, endurance activities, gymnastics, weightlifting, strongman and powerlifting. These exercises are usually combined into a workout of the day (WOD), which is performed at high intensity and in a circular organisation with limited or no recovery time. The most popular benchmarks of CrossFit^®^ are EMOM (every minute to the minute), AMRAP (as many rounds as possible), RFT (rounds for time), chipper (a series of exercises with one round, usually with high repetitions, to be completed in the fastest possible time), ladder (one or more movements where the workload is increased or decreased over time) or tabata (various rounds of high-intensity intervals alternating 20 s of effort with 10 s of rest).

Two recent systematic reviews involving 15,386 CrossFit^®^ practitioners reported that the average prevalence of injury during CrossFit^®^ training over the past 6–12 months ranged from 5% to 74%, with incidence rates ranging from 0.74 to 18.9 per 1000 h of training; the most commonly injured regions were the shoulder (22.6–26%), spine (14.3–24.0%) and knee (12.9–18.0%) [3,4]. Feito, Burrows and Tabb observed higher prevalence values for shoulders (39%) and back (36%) in 3049 CrossFit^®^ practitioners. In addition, their findings highlight that the most common injuries in both male and female CrossFit^®^ participants involve the elbows (12%) and wrists (11%) [5].

Generally, these survey-based research studies identify risk factors for shoulder injuries by comparing data between injured and uninjured CrossFit^®^ athletes. The survey typically asked about risk factors associated with the number of years of participation [4,6], athletes’ weekly training hours [5,6], weekly training frequency [4,5,6], presence of previous injuries [4,7], competitive participation [4], anthropometric characteristics [4,6] and gender [4,6].

Shoulder injuries are the result of the complexity and extreme demands of the WOD, which is often performed with high loads and improper technique [3,5,7,8,9,10]. Shoulder injuries are also caused by the technical execution of exercises that require a high range of motion (ROM) and stability of the joint complex. In gymnastics exercises, such as rope climbs, pull-ups and ring muscle-ups, the shoulder joint supports an extreme ROM (flexion and abduction) and the pull of the athlete’s body weight, while in weightlifting exercises, such as the overhead squat, push jerk, snatch, and clean and jerk, this joint is exposed to the pushing force and the high ROMs of external rotation, abduction and flexion [3,4].

Weightlifting movements often predispose the athlete to upper-limb injuries, especially shoulder and wrist injuries, in CrossFit^®^ and other sports [4]. The clean, power clean or hang power clean are technically complex movements derived from the clean and jerk [11]. These exercises require the neuromuscular system’s ability to develop a series of high-intensity muscle contractions to accelerate the barbell [12]. In addition, CrossFit^®^ athletes require adequate upper-limb flexibility for the phases of the movement that require a high ROM [13,14,15]. If an athlete is tight in the wrists, elbows and shoulders, receiving the bar on the front shoulders and clavicles (catch phase of the clean movement) can cause two technical problems: the small and ring fingers pop off the bar, and the barbell lands on an anterior area of the deltoid muscle, possibly even on the sternum. Consequently, both technical errors lead to repetitive trauma/stress/overload to the joints and injury [8,16,17,18,19].

Strength and conditioning coaches and athletic trainers should know the reference values (ROM cut-points) for each upper-limb movement (wrist, elbow and shoulder) required in weightlifting exercises in order to implement a flexibility program that allows for correct technical movement and reduces the risk of injury. Achieving the ROM cut-point values required to achieve optimal technique in weightlifting movements should be the goal of flexibility training for CrossFit^®^ athletes. This contribution to strength and conditioning and health training can provide a complete understanding of what ROM is required in each joint to successfully perform these weightlifting movements [20]. However, what the optimal ROM of the upper limbs needs to be in order for athletes to perform the clean movement in CrossFit^®^ efficiently and safely has not yet been investigated [21]. 

Therefore, the aim of this study was to determine the optimal upper-limb ROM profile for the catch phase of the clean movement and to identify the key ROMs for performing the catch phase of the clean movement in CrossFit^®^ athletes. 

The hypothesis of the study was that CrossFit^®^ athletes who correctly execute the catch phase of the clean movement will have a greater ROM in the shoulder, elbow and wrist than athletes who do not execute the catch phase of the clean movement correctly. It was also hypothesised that external rotation of the shoulder, pronation of the elbow and extension of the wrist would predict correct execution of the catch phase of the clean movement.

## 2. Materials and Methods

### 2.1. Study Design

A case-control study embedded in a prospective cohort study was conducted to determine the optimal upper-limb training ROM for CrossFit^®^ athletes who properly applied the catch phase of the clean movement (CPCM) technique. This study followed the Strengthening the Reporting of Observational Studies in Injury and Illness in Sports Guidelines and Checklist (STROBE-SIIS) [22]. The study design thus took into account all the recommendations of the Declaration of Helsinki and was approved by the Ethics and Science Committee of the University of Murcia (ID: 1702/2017). 

A sample of twenty-six CrossFit^®^ athletes aged 20–36 years was recruited from a CrossFit^®^ gym. 

Instruction in the testing procedure of the ROM and 80% 1RM of the CPCM test (familiarisation session) took place one week prior to the testing procedure at the CrossFit^®^ gym. A warm-up and dynamic flexibility were performed before the ROM and CPCM tests.

The exposure corresponded to upper-limb ROM values at the beginning of the sports season: nine passive shoulder (*n* = 4), elbow (*n* = 3) and wrist (*n* = 2) ROMs. Three measurements were taken for each ROM test and the median was used for the subsequent statistical analysis. Information was also collected regarding confounding variables, such as age, anthropometric measures, CrossFit^®^ experience and the athlete’s level of competition. For seven months (events), the athletes performed their usual CrossFit^®^ training and competitions (Table 1). Finally, a prospective measurement of the CPCM technique was performed after 7 months (outcome). Later, the athletes with the correct (CT group or case sample) or incorrect (IT group or control sample) technique of the CPCM (80% 1RM) were classified. The CPCM technique was qualitatively assessed three times.

All athletes were instructed not to perform strenuous exercise 48 h prior to the assessment and were injury free at the time of the assessment. Athletes were asked to wear light clothing (sports top and shorts) and no shoes. Testing was conducted in an independent room of the CrossFit^®^ gym under stable environmental conditions of 25 °C.

### 2.2. Participants

All CrossFit^®^ gym athletes were invited to participate in the study on a voluntary basis. Twenty-six CrossFit^®^ athletes (age: 22–36 years, body mass: 72.25 ± 14.09 kg, body height: 171.45 ± 9.12 cm, body mass index: 24.40 ± 3.17 kg/m^2^) voluntarily participated in this study. General inclusion criteria that applied to both samples were that they were actively training for CrossFit^®^ and had at least two years of CrossFit^®^ training experience. Those athletes who had upper limb or back pain or injury during the study or six months prior to the start of the study were excluded. 

Specific criteria for cases were the correct performance of the CPCM test and participation in at least one Spanish CrossFit^®^ Open. A double-blind method was developed in which the athletes and examiners did not know which participants were assigned to each cohort (CT group (cases) or IT group (control)). All athletes were informed of the benefits and risks of this study and signed a written informed consent form prior to testing, which was approved by the institution. The power of the sample size for this study was analysed as described in the statistical analysis section. 

### 2.3. Procedure Study

#### 2.3.1. Age, Anthropometric and CrossFit^®^ Training Data

First, weights and heights were measured with a mobile stadiometer (Seca 799; Seca Ltd., Hamburg, Germany) with accuracies of 0.1 cm and 0.5 kg, respectively. The body mass index was calculated from the body mass and height by dividing the body mass (kg) by height (m) squared. A correction of 0.5 kg was made for the clothing weight. Next, the athletes were asked about their age, CrossFit^®^ experience (years of training experience, training frequency per week, training duration and participation in the Spanish CrossFit^®^ Open) and injury history.

#### 2.3.2. Upper-Limb ROM Profile Assessment

**Figure 1:**
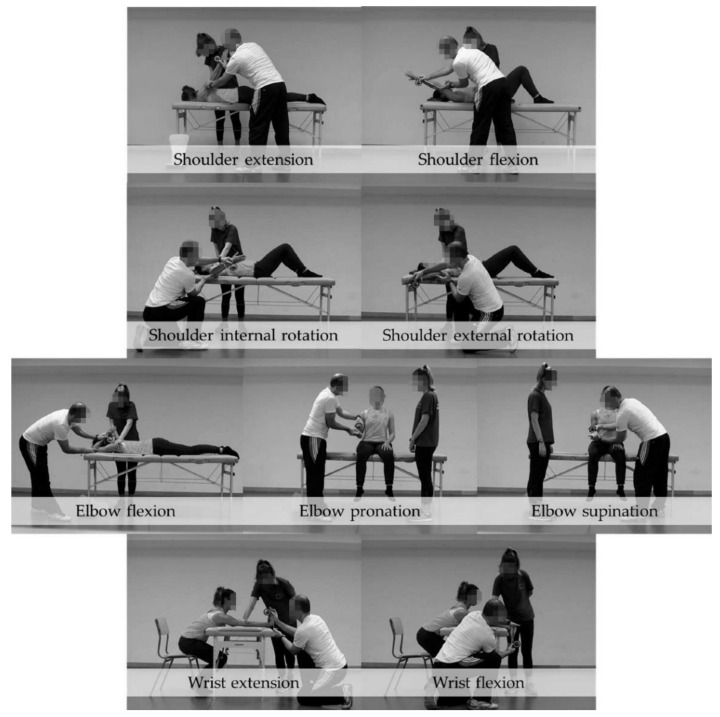
Range of motion assessment tests in the present study.

Aerobic and dynamic stretching exercises were performed before the ROM test. The main passive ranges of motion of the shoulder (extension (SE), flexion (SF), external rotation (SER) and internal rotation (SIR)), elbow (flexion (EF) with shoulder flexion at 180°, pronation (EP) and supination (ES)) and wrist (flexion (WF) and extension (WE) with elbow extension at 0°) were measured with an inclinometer (ISOMED, Inc., Portland, OR, USA). The ROM tests were performed in a random order that was decided using the software http://www.randomizer.org. The inclinometer was calibrated to 0° with either the vertical (shoulder extension and flexion) or horizontal (elbow pronation, shoulder external rotation and internal rotation) before the start of the test session. Elbow flexion and wrist extension were calibrated with the position of the arm and forearm, respectively (Figure 1). 

The movements measured were chosen because they are used by CrossFit^®^ athletes in the clean movement. Of the phases of this movement, the catch phase requires a greater range in the upper limbs. The ROM was measured using the ROM-SPORT method [23,24]. Two athletic trainers with at least 10 years of experience in musculoskeletal assessment conducted the data collection for this study. They had the same competencies in assessing ROMs. The principal examiner performed the ROM tests while the assistant examiner checked the compensatory movements and recorded the data. Based on the minimum detectable change at a 95% confidence interval (MDC_95%_), a third measurement was taken if a difference of more than 6° was detected between the two measurements [23]. Each measurement was taken once before all measurements were repeated. The ROM-SPORT battery was selected in this study for its reliability and validity based on sports experience and biomechanical knowledge [24,25]. 

The intraexaminer reliability of ROM measurements at the shoulder, elbow and wrist using an inclinometer (ISOMED, Inc, Portland, OR, USA) was established previously [23,26]. The test–retest reliability (10 physically active subjects, 2 assessment sessions 24 h apart) in a preliminary double-blind study ranged from 0.90 (shoulder ROM) to 0.96 (elbow and wrist ROM) for ICCs and from 4° (elbow and wrist ROM) to 7° (shoulder ROM) for MDC_95%_. 

#### 2.3.3. Clean Movement Technique Assessment

Each athlete was asked to perform a general (aerobic exercise and dynamic stretching exercises) and specific warm-up for the clean movement before approaching the target load (80% 1RM) of the test. This load is considered optimal for stabilising the technical gesture of an Olympic movement [27,28]. 

**Figure 2:**
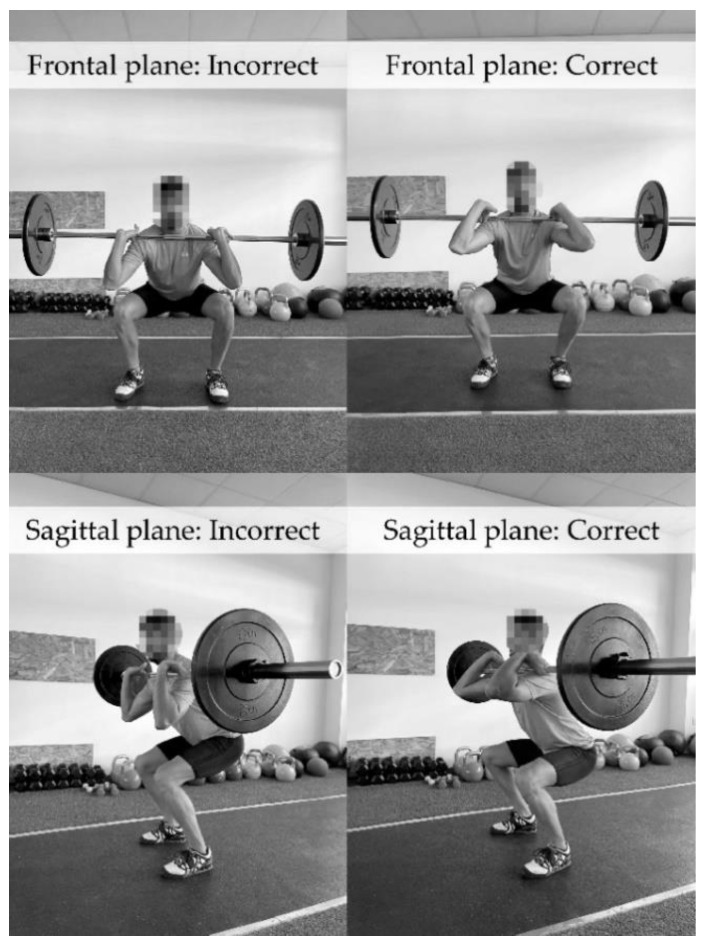
Test procedure for the catch phase of the clean movement.

After a 5 min break, the clean movement was recorded in the frontal and sagittal planes by an Olympic movement expert using a SONY FDRA-X33 4K Ultra HD digital video camera (Sony Europe B.V., Weybridge, Surrey, United Kingdom). Later, the CPCM technique was analysed using the Kinovea Version 0.9.5 software (Joan Charmant & Contributors, Bordeaux, France). The athletes were divided by the expert into a CT group and an IT group according to the technique used for the CPCM. The expert, who was a graduate of physical activity and sport sciences with a weightlifting coach level 1 qualification, focused their examination on the catch position of the clean, which is the phase with greater technical complexity and greater demands on the upper limbs’ ROM. The expert (Figure 2) considered a “correct execution” had occurred when the athlete received the barbell in the front squat position on the front of the shoulders with the elbows pointing forward, the upper arm almost parallel to the floor (≥80° shoulder flexion) and a relaxed grip [29]. In the frontal plane, the grip of the barbell should be done with the hands and fingers and be half a fist width to one fist width from the shoulder, and the elbows should be no more than shoulder width apart [30]. 

### 2.4. Statistical Analysis

The sample size was calculated post hoc with input parameters (effect size = 1.50, alpha = 0.05, power = 0.94) to provide a difference between two independent (two groups) tests (software package G*Power version 3.1.9.4 (Heinrich Heine-Universität Düsseldorf, Düsseldorf, Germany)). The effect size was calculated from the average of the ROM variables (shoulder external rotation and wrist extension, elbow at 0° ROM; Table 2) with significant differences between groups (CT group vs. IT group).

Statistical analyses were performed using the JASP version 0.14.01 software (JASP -Team University of Amsterdam, Amsterdam, The Netherlands). 

Due to the final sample size, it was decided to use Bayesian statistics instead of frequentist statistics. Bayesian inference was recently proposed as an alternative that is more robust than traditional frequentist statistics (based on confidence intervals and *p*-values) for hypothesis testing. This method is based on quantifying the relative degree of evidence for two competing hypotheses [31,32], namely, the null hypothesis (H0, B_01_) versus the alternative hypothesis (H1, B_10_), using the Bayes factor (BF_01_–BF_10_). Hypotheses H1 and H0 refer to the probability that the comparison of the evaluated variables is different and equal, respectively.

Normality was confirmed with the Shapiro–Wilk test. Non-normally distributed data showed a Gaussian distribution after logarithmic transformation. All continuous data are expressed as mean ± standard error with a 95% confidence interval.

Differences between dominant and non-dominant limbs in the shoulder, elbow and wrist ROMs were compared using the Bayesian Wilcoxon signed-rank test. The Bayesian Mann–Whitney U test was performed to detect differences between the sexes (male group vs. female group; Table 1) and the CPCM techniques (CT group vs. IT group; Table 2).

The BF_10_ was interpreted using the evidence categories proposed by Lee and Wagenmakers: <1/100—extreme evidence for H0, from 1/100 to 1/30—very strong evidence for H0, from 1/30 to 1/10—strong evidence for H0, from 1/10 to 1/3—moderate evidence for H0, from 1/3 to 1—anecdotal evidence for H0, from 1 to 3—anecdotal evidence for H1, from 3 to 10—moderate evidence for H1, from 10 to 30—strong evidence for H1, from 30 to 100—very strong evidence for H1 and >100—extreme evidence for H1 [33]. The models that showed at least strong evidence with a percentage error >10 were considered robust enough to describe the main effects. The median and central 95% credibility interval (CI) of the posterior distribution of the standardised effect size (δ) were also calculated for each of the between-group comparisons. Magnitudes of the posterior distribution of the standardised effect size were classified as follows [34]: very large (2.0–4.0), large (1.2–2.0), moderate (0.6–1.2), small (0.2–0.6) and trivial (<0.2).

Receiver operating characteristic (ROC) analyses were performed using the open-source statistical software Jamovi version 1.6.23 (The Jamovi Project, Sydney, Australia) to determine the optimal cut-off values for the ROMs assessed [35]. The predictive ability of the identified predictors was calculated using the area under the curve (AUC). The AUC was used to rank the combined sensitivity and specificity as no discrimination (AUC < 0.50), poor (0.50 ≥ AUC < 0.80), acceptable (0.70 ≥ AUC < 0.80), excellent (0.80 ≥ AUC < 0.90) and outstanding (0.90 ≥ AUC < 1.00) [36]. Second, the optimal cut-off value (the value that provided the highest discriminatory ability) that maximised the ratio between sensitivity (true positive rate) and specificity (true negative rate) was determined using the Youden index, i.e., the optimal cut-off value that provides the best discriminatory ability between athletes who correctly performed the CPCM technique and those who did not [37]. In addition, the positive predictive value (PPV) and negative predictive value (NPV) were calculated. 

The correlation between the ROM (low versus high for the optimal cut-off value) and the execution of the CPCM was determined using Pearson’s chi-square statistic.

## 3. Results

Six athletes were excluded from the preliminary sample because they showed shoulder pain during the assessment session. Finally, a sample of 20 athletes was selected for the study. The power of the sample size was 0.83 (effect size = 1.25, alpha = 0.05, CT group = 8, IT group = 12).

The characteristics of the athletes who met the inclusion and exclusion criteria of this study are shown in Table 1. Significant differences between the sexes were found regarding age, body mass, body height and body mass index (BF_10_ ≥ 20.55, 95% credibility interval ≥ 1.39 (large)).

There were differences (BF_10_ ≥ 12.74, 95% credibility interval ≥ 1.27 (large)) between the CT and IT groups in terms of training experience, training frequency, training duration, SER ROM and WE ROM.

**Figure 3:**
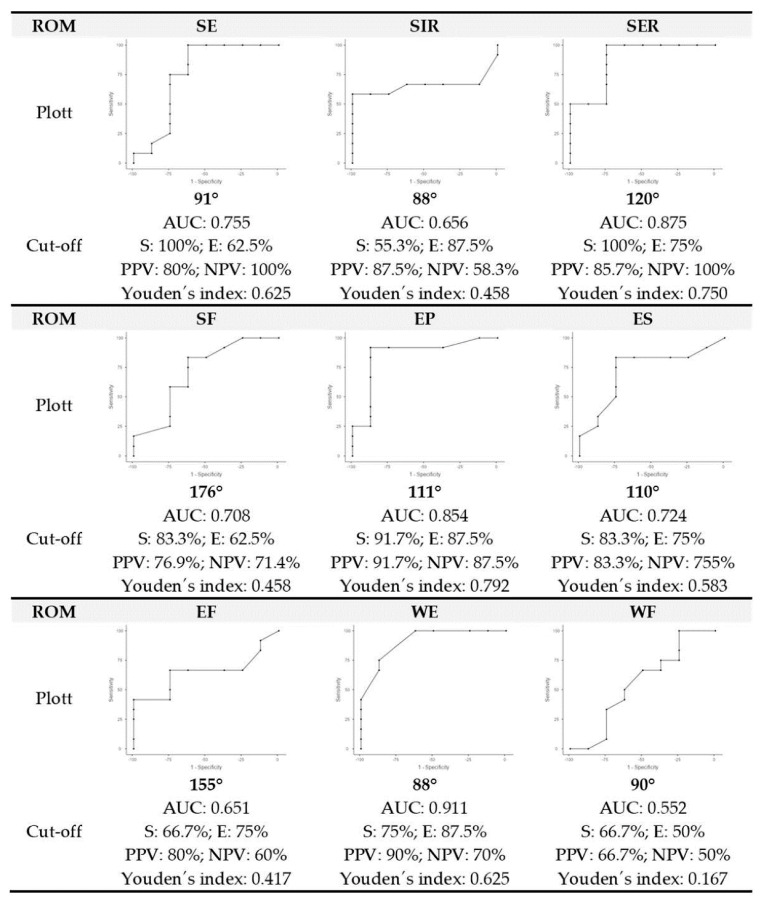
Results of the cut-off points that discriminated the correct technique in the catch phase of the clean movement. ROM: range of motion; AUC: area under the curve; S: sensibility; S: specificity; SE: shoulder extension; SER: shoulder external rotation; SIR: shoulder internal rotation; SF: shoulder flexion; EP: elbow pronation; ES: elbow supination; EF: elbow flexion; WF: wrist flexion; WE: wrist extension; PSV: positive predictive value; NPV: negative predictive value.

The maximum value of Youden’s index determined the optimal cut-off points for the SE, SIR, SER, SF, EP, ES, EF, WF and WE ROMs at 93°, 88°, 123°, 177°, 112°, 111°, 150°, 90° and 100°, respectively (Figure 3). However, the SER (123°), EP (112°) and WF (90°) ROMs were the most decisive movements for an athlete to achieve an optimal CPCM (area under the curve ≥ 0.854). The probability (positive predictive value) that an athlete with values of ≥123° for SER, ≥112° for EP and ≥90° for WF would achieve the correct execution of the CPCM was ≥75%. 

The chi-square test revealed that with a SER ROM ≥ 123° (*χ*^2^(20) = 12,857, *p* = 0.000, *ղ*^2^ = 0.802), EP ROM ≥ 112° (*χ*^2^(20) = 12,535, *p* = 0.000, *ղ*^2^ = 0.792) and WF ROM ≥ 90° (*χ*^2^(20) = 12,857, *p* = 0.000, *ղ*^2^ = 0.802) was associated with an adequate CPCM technique.

## 4. Discussion

References to clean kinematics were published previously [1,29,38,39]. However, to date, the scientific literature has not quantitatively described the maximum passive ROM required to perform the clean movement efficiently and safely, especially the catch phase. This phase requires muscle extensibility and a specific ROM to receive and stabilise the barbell at the front and upper part of the shoulder girdle in a deep front squat. The main finding was that the optimal ROM profile required to perform the CPCM correctly was determined to be 93°, 88°, 123°, 177°, 112°, 111°, 150°, 90° and 100° for the SE, SIR, SER, SF, EP, ES, EF, WF and WE ROMs, respectively. The optimal ROM profile was determined using a classification prediction model. This classification sets a threshold (cut-off) with sufficient discriminatory power to perform the CPCM technique correctly. The probability of success of an optimal ROM ranged from 65.1% to 91.1% (AUC = 0.552 to 0.911) for the nine movements studied. The ROM can be used to predict performance in strength sports [40]. Previous studies showed that higher ROM values allow for biomechanical benefits, such as better physical performance through the use of strength for longer periods [41,42,43,44,45] and higher technical quality of movement [7,21,46]. Once an athlete has established the ROM profile of the technical movement, athletes can focus on the next goals, namely, technical efficiency and load mobilisation, which are considered the key elements to optimising athletic performance in weightlifting movements [47,48].

Other biomechanical benefits enabled by an optimal ROM are the reduction of stress and joint loads during the CPCM, which can consequently minimise degenerative changes in joint tissues observed in overhead athletes [21,49]. As a result, the biological capacity of the joint tissue remains stable [50,51]. In addition, a specific ROM can help prevent the occurrence of compensatory movements [52,53], muscular imbalances [54,55] and inadequate clean executions [7,21,46], which are considered to be factors and mechanisms of the frequent injuries in CrossFit^®^ [7,21,46,55]. In this sense, the incidence of traumatic injuries (acromioclavicular and sternoclavicular dislocation, sternoclavicular abscess) or overuse injuries (osteophytes on the dorsal side of the wrist at the distal radioulnar joint, stress fractures in the radius), which are common in those who habitually perform the clean movement [56], could be reduced if the athlete had the ROM values described in this study. To avoid these injuries, Morton, Whitehead, Brinkert and Caine [57] suggested performing strength exercises with the full ROM of the joint as a technical element to avoid ROM restrictions, but with moderate loads. In contrast, several authors show that the repeated performance of exercises with a full ROM and heavy loads (e.g., bench press, shoulder press, dumbbell fly, etc.) contributes to the development of overuse injuries, such as dynamic shoulder instability, subacromial syndrome and rotator cuff tears [58,59]. Therefore, we recommend other more efficient and safer interventions to improve muscle extensibility and ROM, which are required during strength exercises, such as stretching [60,61,62] and foam rolling [61,63].

To our knowledge, only five previous cross-sectional studies investigated ROMs in strength sports [55,64,65,66,67]. ROMs are specific to the sport and the characteristics of the sample studied, such as age, years of experience and level of competition [68]. It appears that the physical and technical demands of CrossFit^®^ favour positive adaptations of the ROM compared with athletes of other strength sports, such as powerlifting [64,65], bodybuilding [66], Olympic weightlifting [67], and fitness with weights and machines [55]. In the CrossFit^®^ athletes, higher values of their ROM were observed in all assessed movements (SIR ROM: 89° in CrossFit^®^, 43.1° and 57.4° in powerlifting, 60.5° in bodybuilding; SER ROM: 107° in CrossFit^®^, 78.2° and 77.9° in powerlifting, 104.7° in bodybuilding; SE ROM: 78° in CrossFit^®^, 10.5° and 40.8° in powerlifting; EF ROM: 153° in CrossFit^®^, 130.5° in powerlifting; EP ROM: 99° in CrossFit^®^, 83.5° in powerlifting; WE ROM: 92° in CrossFit^®^, 46.5° in powerlifting) with the exception of SF ROM (173° in CrossFit^®^, 94.8° and 156.2° in powerlifting, 160.3° and 183.6° in bodybuilding, 202° in Olympic weightlifters). A possible explanation for this finding could be that CrossFit^®^ athletes perform overhead exercises more frequently than powerlifters, bodybuilders, and weight and machine fitness practitioners. In addition, CrossFit^®^ functional exercises are performed with a complete ROM of the technical gesture in order to be counted by the competition judges. However, bodybuilders and weight fitness practitioners do not usually use overhead and wide ROM exercises (with the exception of the shoulder press, pull-down and French press), as their main goal is aesthetics and muscle hypertrophy [55,66]. Like competitive powerlifting, it focuses mainly on three exercises, the bench press, deadlift and squat [69]. The results of these studies were inconsistent, as the sample size (ranging from 10 to 60 participants), the age range from 20 to 62 years, the experience in practicing their sport from 3 months to 25 years and the weekly training frequency from 2 to 5 days/week varied greatly. Other factors that may affect the values of the ROM are the type of exercise, with higher values seen for passive exercise than active exercise [60]; the starting position of the athlete (seated, prone, supine or lateral), with lower values observed in the decubitus position than in the seated position due to better stabilisation and control of compensatory movements when lying on a table [70]; the use [65,66] or not [64] of warming up before assessment; and competition experience, where a higher ROM was correlated with athletes with higher competition levels in the same sport [71]. However, perhaps the main reason for the observed discrepancies in the shoulder ROM values (SF ROM: 94.8° in powerlifting vs. 173° in CrossFit^®^, 94.8° and 156.2° in powerlifting, 160.3 and 183.6° in bodybuilding; SIR ROM: 43.1° and 57.4° in powerlifting vs. 89° in CrossFit^®^, 57.4° in powerlifting, 60.5° in bodybuilding; SER ROM: 78.2° and 77.9° in powerlifting vs. 107° in CrossFit^®^, 104.7° in bodybuilding; SE ROM: 10.5° in powerlifting vs. 78° in CrossFit^®^, 40.8° in bodybuilding) were likely due to the angle assessment procedure used in each study. Fixation and control of the scapula may be the methodological variable that has the greatest influence on the ROM [65], as it significantly limits the SF and SE ROMs [72].

As for the second objective of this study, SER, EP and WE were the most important ROMs for the correct execution of the CPCM with a probability of success of more than 85%. These facts were previously observed in the difference of the ROM means between the two groups (CT group vs. IT group) according to the CPCM, where differences were found in these three ROMs. The kinematic description of the clean movement remarkably shows these three ROMs [29,30].

The increased flexibility of the shoulder internal rotators, the elbow supinators and the wrist flexors could be due to the fact that these muscles are trained using a comparatively large ROM during the CPCM. Eccentric training is an effective method of increasing muscle flexibility [73]. However, if an athlete has tight shoulder internal rotators, elbow supinators and wrist flexors, performing the clean with loads to correctly perform the CPCM will result in repetitive trauma/stress/overload to the joints and injury [16,17]. The injuries often occur after eccentric contractions, when the muscle is under tension and stretched with a force greater than the force generated by the muscle. Impaired activation and structural disruption of the sarcomere are possible mechanisms responsible for eccentrically induced muscle injuries [74]. Heavy eccentric exercise also causes early loss of the cytoskeletal protein desmin and a loss of cellular integrity that manifests itself in damage to the sarcolemma [75]. Changes in the ultrastructure of skeletal muscle, such as the disarray of individual lines and bands in the sarcomere, vascular degeneration of mitochondria, swelling of the sarcoplasmic reticulum and disordered arrangement of myofibrils, may be caused by eccentric training [76]. Therefore, the use of a flexibility program in complementary sessions is recommended to increase the extensibility of the muscles and ROM. This type of intervention is as efficient or more efficient but much safer than strength exercises with a load and maximum ROM. It is also recommended to perform flexibility exercises such as dynamic flexibility and active stretching during warm-up and passive stretching during cool-down. Athletic trainers need to select exercises (e.g., shoulder: sleeper stretch, bully extension bias, reverse wall slide; elbow: banded front rack; wrist: wrist extension) that safely and effectively move the muscle origin and insertion. The stretching load should follow the international recommendations for flexibility exercises, e.g., 2–3 sets of 30 s and a medium stretch tolerance intensity.

On the other hand, the age, anthropometric data and sport experience of the CrossFit^®^ athletes differed according to the CPCM. Athletes who had more years of training experience and training volume (training frequency per week and training duration) were found with strong robustness to correctly execute the CPCM. Furthermore, body mass and body mass index also have a moderate influence on performance in the CPCM. It is possible that low values of both body composition variables are associated with higher athletic performance [77,78]. With regard to gender, differences were found in age, body mass and body mass index. Male athletes had higher values than female athletes. These results are consistent with previous studies that have examined age [79], body mass [79,80,81] and body mass index [79,81] in strength sports such as powerlifting, CrossFit^®^ and bodybuilding. The greater flexibility of female athletes predisposes them to the correct execution of the CPCM. With regard to ROM, moderately higher values (B_10_ = 3 to 10) were found in female athletes than in male athletes for SER and EP ROMs. Therefore, the differences in ROM by sex were not sufficiently robust to achieve the study’s aim of separating male and female athletes. However, these moderate differences in the SER and EP ROMs in favour of the female athletes and even the higher values in the rest of the ROMs can be considered as an important factor for the correct performance of the CPCM. In this sense, the percentage of success observed in this study regarding the correct execution of the CPCM was 80% female athletes versus 25% male athletes. Previous work investigating the upper-limb ROM in strength athletes did not develop this study objective [55,64,65,66,67].

Although CrossFit^®^ promotes a progressive training program and safe exercise execution technique, improving body composition and physical fitness, including flexibility training (SER, EP and WE ROMs) may improve the performance of the clean and reduce injury in CrossFit^®^ athletes.

## 5. Conclusions

The optimal upper-limb profile that predicted correct execution of the catch phase of the clean movement was 78° in shoulder extension, 173° in shoulder flexion, 107° in shoulder external rotation, 89° in shoulder internal rotation, 153° in elbow flexion, 99° in elbow pronation and 92° in wrist extension. It was found that three ROM for the SER, EP and WE ROMs are associated with the proper execution of the catch phase of the clean movement with a probability of success of more than 85%. CrossFit^®^ athletes should improve the upper-limb ROM profile, technical training (refining clean movement patterns) and a minimum amount of training experience prior to increasing loads on the cleans. Further studies are warranted to determine whether improving flexibility for upper-limb ROMs may improve the clean technique and performance.

## Figures and Tables

**Table 1:** Athletes’ descriptive characteristics (mean ± standard deviation) according to sex.

Variables	Male (*n* = 12)	Female (*n* = 8)	Bayesian Factor	δ (95% Credible Interval)	Evidence	Total (*n* = 20)
Age (y)	32.67 ± 3.31	27.25 ± 2.71	25.59	1.45 (0.39, 2.57)	Strong B_10_	30.50 ± 4.06
Body mass (kg)	80.42 ± 12.07	60.00 ± 4.93	84.65	1.77 (0.62, 2.95)	Very strong B_10_	72.25 ± 14.09
Body height (cm)	175.50 ± 9.45	165.38 ± 3.85	4.98	1.01 (0.09, 2.04)	Moderate B_10_	171.45 ± 9.12
Body mass index (kg/m^2^)	26.06 ± 2.98	21.91 ± 1.22	20.55	1.39 (0.35, 2.51)	Strong B_10_	24.40 ± 3.17
Training experience (y)	2.92 ± 1.83	3.13 ± 1.89	2.44	−0.06 (−0.82, 0.67)	Anecdotal B_01_	3.00 ± 1.81
Months training per year (mth) *	11.67 ± 0.49	11.50 ± 0.53	2.07	0.21 (−0.51, 1.01)	Anecdotal B_01_	11.60 ± 0.50
Training frequency per week (day) *	3.33 ± 1.30	3.50 ± 1.20	2.37	−0.11 (−0.87, 0.62)	Anecdotal B_01_	3.40 ± 1.23
Training session duration (min) *	70.00 ± 14.77	71.88 ± 15.10	2.40	−0.09 (−0.85, 0.64)	Anecdotal B_01_	72.35 ± 14.53
Shoulder extension (degree)	84.08 ± 8.67	88.88 ± 12.51	1.73	−0.31 (−1.14, 0.42)	Anecdotal B_01_	86.00 ± 10.34
Shoulder internal rotation (degree)	91.92 ± 9.02	87.38 ± 9.12	1.63	0.33 (−0.39, 1.18)	Anecdotal B_01_	90.10 ± 9.11
Shoulder external rotation (degree)	111.67 ± 9.77	124.63 ± 11.92	3.73	0.92 (−1.94, −0.04)	Moderate B_10_	116.85 ± 12.25
Shoulder flexion (degree)	173.08 ± 3.63	176.88 ± 4.36	1.73	−0.698 (−1.66, 0.11)	Anecdotal B_10_	174.60 ± 4.27
Elbow pronation (degree) *	104.25 ± 7.39	113.25 ± 2.43	8.64	−1.16 (−2.23, −0.19)	Moderate B_10_	107.85 ± 7.36
Elbow supination (degree)	107.58 ± 5.05	109.00 ± 5.40	2.19	−0.18 (−0.97, 0.54)	Anecdotal B_01_	108.15 ± 5.10
Elbow flexion (degree)	155.25 ± 8.29	155.75 ± 6.92	2.46	−0.041 (−0.79, 0.69)	Anecdotal B_01_	155.45 ± 7.58
Wrist extension, elbow at 0° (degree) *	84.33 ± 7.56	91.38 ± 5.73	2.03	−0.75 (−1.71, 0.08)	Anecdotal B_10_	87.15 ± 7.60
Wrist flexion, elbow at 0° (degree)	90.92 ± 4.85	93.00 ± 6.63	2.00	−0.23 (−1.04, 0.49)	Anecdotal B_01_	91.75 ± 5.56

* Variables that were changed using a logarithmic transformation.

**Table 2:** Demographic, anthropometric, sports and range of motion data differences of the CrossFit^®^ athletes according to the catch phase of the clean movement (CPCM) technique.

Variables	Incorrect CPCM (*n* = 12)	Correct CPCM (*n* = 8)	Bayesian Factor	δ (95% Credible Interval)	Evidence
Age (y)	31.83 ± 3.93	28.50 ± 3.59	1.37	0.62 (−1.16, 1.56)	Anecdotal B_10_
Body mass (kg)	78.17 ± 13.50	63.38 ± 10.14	3.56	0.91 (0.03, 1.92)	Moderate B_10_
Height (cm)	173.75 ± 9.39	168.00 ± 8.02	1.25	0.44 (−0.30, 1.32)	Anecdotal B_01_
Body mass index (kg/m^2^)	25.79 ± 3.21	22.31 ± 1.65	4.67	0.99 (0.08, 2.02)	Moderate B_10_
Training experience (y)	2.08 ± 1.44	4.38 ± 1.41	19.08	−1.38 (−2.48, −0.34)	Strong B_10_
Months training per year (mth)	11.58 ± 0.51	11.63 ± 0.52	2.45	−0.05 (−0.81, 0.68)	Anecdotal B_01_
Training frequency (per week)	2.75 ± 0.87	4.38 ± 1.06	12.74	−1.27 (−2.35, −0.26)	Strong B_10_
Training duration (min) *	62.5 ± 8.66	83.13 ± 12.08	67.03	−1.70 (−2.87, −0.57)	Very strong B_10_
Shoulder extension (degree)	81.83 ± 6.60	92.25 ± 12.14	2.89	−0.85 (−1.85, 0.01)	Anecdotal B_10_
Shoulder internal rotation (degree)	88.50 ± 11.24	92.50 ± 3.96	1.80	−0.29 (−1.12, 0.43)	Anecdotal B_01_
Shoulder external rotation (degree)	109.83 ± 7.03	127.38 ± 10.85	69.70	−1.71 (−2.89, −0.58)	Very strong B_10_
Shoulder flexion (degree)	173.33 ± 3.45	176.50 ± 4.90	1.07	−0.54 (−1.45, 0.22)	Anecdotal B_10_
Elbow pronation (degree) *	104.83 ± 7.46	112.38 ± 4.57	3.13	−0.85 (−1.84, 0.01)	Moderate B_10_
Elbow supination (degree)	106.58 ± 5.00	110.50 ± 4.57	1.15	−0.57 (−1.49, 0.21)	Anecdotal B_10_
Elbow flexion (degree)	153.67 ± 8.39	158.13 ± 5.64	1.37	−0.40 (−1.27, 0.33)	Anecdotal B_01_
Wrist extension, elbow at 0° (degree) *	83.33 ± 6.77	92.88 ± 4.70	13.20	−1.28 (−2.36, −0.27)	Strong B_10_
Wrist flexion, elbow at 0° (degree)	91.25 ± 5.05	92.50 ± 6.55	2.30	−0.14 (−0.91, 0.59)	Anecdotal B_01_

* variables that changed in logarithmic transformation.

## Data Availability

The data associated with the study are not publicly available but are available from the corresponding author upon reasonable request.

## References

[1] CrossFit L. About Affiliation. https://www.crossfit.com/gyms.

[2] CrossFit L. What Is CrossFit?. https://www.crossfit.com/what-is-crossfit.

[3] Hech Dominski F., Cristina Siqueira T., Teixeira Serafim T., Andrade A. (2018). Injury profile in CrossFit practitioners: Systematic review. Fisioter. Pesqui..

[4] Rodríguez M., García-Calleja P., Terrados N., Crespo I., Del Valle M., Olmedillas H. (2022). Injury in CrossFit^®^: A Systematic Review of Epidemiology and Risk Factors. Physician Sportsmed..

[5] Feito Y., Burrows E., Tabb L. (2018). A 4-year analysis of the incidence of injuries among CrossFit-trained participants. Orthop. J. Sports Med..

[6] Montalvo A., Shaefer H., Rodriguez B., Li T., Epnere K., Myer G. (2017). Retrospective injury epidemiology and risk factors for injury in CrossFit. J. Sports Sci. Med..

[7] Summitt R., Cotton R., Kays A., Slaven E. (2016). Shoulder injuries in individuals who participate in CrossFit training. Sports Health.

[8] Weisenthal B., Beck C., Maloney M., DeHaven K., Giordano B. (2014). Injury rate and patterns among CrossFit athletes. Orthop. J. Sports Med..

[9] Hak P., Hodzovic E., Hickey B. (2013). The nature and prevalence of injury during CrossFit training. J. Strength Cond. Res..

[10] Tafuri S., Salatino G., Napoletano P., Monno A., Notarnicola A. (2018). The risk of injuries among CrossFit athletes: An Italian observational retrospective survey. J. Sports Med. Phys. Fit..

[11] Suchomel T., Comfort P., Stone M. (2015). Weightlifting Pulling Derivatives: Rationale for Implementation and Application. Sports Med..

[12] Suchomel T., Comfort P., Lake J. (2017). Enhancing the force-velocity profile of athletes using weightlifting derivatives. Strength Cond. J..

[13] Comfort P., Williams R., Suchomel T. (2017). A comparison of catch phase force-time characteristics during clean derivatives from the knee. J. Strength Cond. Res..

[14] Stone M., Pierce K., Sands W., Stone M. (2006). Weightlifting: A brief overview. Strength Cond. J..

[15] Fry A., Ciroslan D., Fry M., LeRoux C., Schilling B., Chiu L. (2006). Anthropometric and performance variables discriminating elite American junior men weightlifters. J. Strength Cond. Res..

[16] Aasa U., Svartholm I., Andersson F., Berglund L. (2017). Injuries among weightlifters and powerlifters: A systematic review. Br. J. Sports Med..

[17] Strömbäck E., Aasa U., Gilenstam K., Berglund L. (2018). Prevalence and consequences of injuries in powerlifting: A cross-sectional study. Orthop. J. Sports Med..

[18] Keogh J., Winwood P. (2017). The Epidemiology of Injuries Across the Weight-Training Sports. Sports Med..

[19] Sprey J., Ferreira T., De Lima M., Duarte A., Jorge P., Santili C. (2016). An epidemiological profile of CrossFit athletes in Brazil. Orthop. J. Sports Med..

[20] Bousquet B., Olson T. (2018). Starting at the Ground Up: Range of Motion Requirements and Assessment Procedures for Weightlifting Movements. Strength Cond. J..

[21] Claudino J., Gabbett T., Bourgeois F., Souza H., Miranda R., Mezêncio B., Soncin R., Cardoso Filho C., Bottaro M., Hernandez A. (2018). CrossFit Overview: Systematic Review and Meta-analysis. Sports Med.-Open-Open.

[22] Bahr R., Clarsen B., Derman W., Dvorak J., Emery C., Chamari K. (2020). International Olympic Committee consensus statement: Methods for Recording and Reporting of Epidemiological Data on Injury and Illness in Sports 2020 (Including the STROBE Extension for Sports Injury and Illness Surveillance (STROBE-SIIS)). Orthop. J. Sports Med..

[23] Cejudo A., Sánchez-Castillo S., Sainz de Baranda P., Gámez J., Santonja-Medina F. (2019). Low Range of Shoulders Horizontal Abduction Predisposes for Shoulder Pain in Competitive Young Swimmers. Front. Psychol..

[24] Cejudo A. (2022). Description of ROM-SPORT I Battery: Keys to Assess Lower Limb Flexibility. Int. J. Environ. Res. Public Health.

[25] Gerhardt J., Cocchiarella L., Lea R. (2002). The Practical Guide to Range of Motion Assessment.

[26] Cejudo A., Abril-Guiote J.E., Igualada-Fernández A., Sainz de Baranda P. (2021). Valoración del riesgo de dolor de hombro en trabajadores de limpieza viaria y recogida de residuos. Proyecto PRE-REFILAB. JUMP.

[27] Kilduff L., Bevan H., Owen N., Kingsley M., Bunce P., Bennett M., Cunningham D. (2007). Optimal Loading for Peak Power Output During the Hang Power Clean in Professional Rugby Players. Int. J. Sports Physiol. Perform..

[28] Cormie P., McCaulley G., Triplett N., McBride J. (2007). Optimal loading for maximal power output during lower-body resistance exercises. Med. Sci. Sports Exerc..

[29] Turner A., Comfort P. (2022). Advanced Strength and Conditioning: An Evidence-Based Approach.

[30] Storey A., Smith H. (2012). Unique Aspects of Competitive Weightlifting. Sports Med..

[31] Doncaster G., Page R., White P., Svenson R., Twist C. (2020). Analysis of Physical Demands During Youth Soccer Match-Play: Considerations of Sampling Method and Epoch Length. Res. Q. Exerc. Sport.

[32] Linke D., Link D., Weber H., Lames M. (2018). Decline in match running performance in football is affected by an increase in game interruptions. J. Sports Sci. Med..

[33] Lee M., Wagenmakers E. (2013). Bayesian Data Analysis for Cognitive Science: A Practical Course.

[34] Batterham A., Hopkins W. (2006). Making meaningful inferences about magnitudes. Int. J. Sports Physiol. Perform..

[35] Thiele C. Cutpointr: Determine and Evaluate Optimal Cutpoints in Binary Classification Tasks [R Package]. https://cran.r-project.org/package=cutpointr.

[36] Hosmer D., Lemeshow S., Sturdivant R. (2013). Applied Logistic Regression.

[37] Youden W. (1950). Index for rating diagnostic tests. Cancer.

[38] Takano B. (1987). Coaching optimal technique in the snatch and the clean and jerk. NSCA J..

[39] CrossFit L. CrossFit Certificate Courses. https://www.crossfit.com/certificate-courses.

[40] Spence A., Helms E., McGuigan M. (2021). Range of Motion Is Not Reduced in National-Level New Zealand Female Powerlifters. J. Strength Cond. Res..

[41] Garhammer J. (1985). Biomechanical Profiles of Olympic Weightlifters. Int. J. Sport Biomech..

[42] Winwood P., Cronin J., Brown S. (2015). A biomechanical analysis of the heavy sprint-style sled pull and comparison with the back squat. J. Sports Sci. Coach..

[43] Hindle B., Lorimer A., Winwood P., Keogh J. (2019). The Biomechanics and Applications of Strongman Exercises: A Systematic Review. Sports Med.-Open.

[44] Bloomquist K., Langberg H., Karlsen S., Madsgaard S., Boesen M., Raastad T. (2013). Effect of range of motion in heavy load squatting on muscle and tendon adaptations. Eur. J. Appl. Physiol..

[45] Silva J., Gomes W., Pecoraro S., Soares E., Magalhães R., Fioravanti G., Baladán R., Lopes C., Marchetti P. (2020). The range of motion of the back squat exercise affects absolute volume load without changing the rating of perceived exertion. Rev. Bras. Cineantropometria Desempenho Hum..

[46] da Costa T., Louzada C., Miyashita G., da Silva P., Sungaila H., Lara P., de Castro A., Ejnisman B., Cohen M., Arliani G. (2019). CrossFit s: Injury prevalence and main risk factors. Clinics.

[47] Chen S., Wu M., Huang C., Wu J., Guo L., Wu W. (2013). The analysis of upper limb movement and EMG activation during the snatch under various loading conditions. J. Mech. Med. Biol..

[48] Ammar A., Riemann B., Masmoudi L., Blaumann M., Abdelkarim O., Hökelmann A. (2018). Kinetic and kinematic patterns during high intensity clean movement: Searching for optimal load. J. Sports Sci..

[49] Khaled E., Ibrahim A. (2013). The effect of development of muscular balance on some dynamic parameters and level of achievement for clean and jerk skill for weightlifters. Ovidius Univ. Ann. Ser. Phys. Educ. Sport/Sci. Mov. HealthScience Mov. Health.

[50] Wilk K., Macrina L., Fleisig G., Aune K., Porterfield R., Harker P., Evans T., Andrews J. (2015). Deficits in glenohumeral passive range of motion increase risk of shoulder injury in professional baseball pitchers: A prospective study. Am. J. Sports Med..

[51] Shanley E., Rauh M., Michener L., Ellenbecker T., Garrison J., Thigpen C. (2011). Shoulder range of motion measures as risk factors for shoulder and elbow injuries in high school softball and baseball players. Am. J. Sports Med..

[52] Kendall F., McCreary E., Provance P., Rodgers M., Romani W. (2005). Muscles: Testing and Function with Posture and Pain.

[53] Lewis J., Green A., Wright C. (2005). Subacromial impingement syndrome: The role of posture and muscle imbalance. J. Shoulder Elb. Surg..

[54] Codine P., Bernard P., Benaim C., Brun V. (1997). Influence of sports discipline on shoulder rotator cuff balance. Med. Sci. Sports Excercices.

[55] Kolber M., Beekhuizen K., Cheng M., Hellman M. (2009). Shoulder joint and muscle characteristics in the recreational weight training population. J. Strength Cond. Res..

[56] Lavallee M., Balam T. (2010). An overview of strength training injuries: Acute and chronic. Curr. Sports Med. Rep..

[57] Morton S., Whitehead J., Brinkert R. (2011). Resistance training vs. static stretching: Effects on flexibility and strength. J. Strength Cond. Res..

[58] Fees M., Decker T., Snyder-Mackler L., Axe M. (1998). Upper extremity weight-training modifications for the injured athlete: A clinical perspective. Am. J. Sports Med..

[59] Kolber M., Corrao M., Hanney W. (2013). Characteristics of anterior shoulder instability and hyperlaxity in the weight-training population. J. Strength Cond. Res..

[60] Ayala F., Sainz De Baranda P. (2013). Calidad metodológica de los programas de estiramiento: Revisión sistemática. Int. J. Med. Sci. Phys. Act. Sport.

[61] Mohr A., Long B., Goad C. (2014). Effect of foam rolling and static stretching on passive hip-flexion range of motion. J. Sport Rehabil..

[62] Decicco P., Fisher M. (2005). The effects of proprioceptive neuromuscular facilitation stretching on shoulder range of motion in overhand athletes. J. Sports Med. Phys. Fit..

[63] Wiewelhove T., Döweling A., Schneider C., Hottenrott L., Meyer T., Kellmann M., Pfeiffer M., Ferrauti A. (2019). A meta-analysis of the effects of foam rolling on performance and recovery. Front. Physiol..

[64] Chang D., Buschbacher L., Edlich R. (1988). Limited joint mobility in power lifters. Am. J. Sports Med..

[65] Gadomski S., Ratamess N. (2018). Range of Motion Adaptations in Powerlifters. J. Strength Cond. Res..

[66] Barlow J., Benjamin B., Birt P., Hughes C., Barlow J., Benjamin B., Birt P., Hughes C. (2002). Shoulder strength and range-of-motion characteristics in bodybuilders. J. Strength Cond. Res..

[67] Beedle B., Jessee C., Stone M. (1991). Flexibility characteristics among athletes who weight train. J. Appl. Sport Sci. Res..

[68] Cejudo A., Moreno-Alcaraz V., Izzo R., Robles-Palazón F.J., Sainz de Baranda P., Santonja-Medina F. (2020). Flexibility in Spanish Elite Inline Hockey Players: Profile, Sex, Tightness and Asymmetry. Int. J. Environ. Res. Public Health.

[69] International Powerlifting Federation Technical Rules. https://www.powerlifting.sport/fileadmin/ipf/data/rules/technical-rules/english/IPF_Technical_Rules_Book_2022_1.pdf.

[70] Kouyoumdjian P., Coulomb R., Sanchez T., Asencio G. (2012). Clinical evaluation of hip joint rotation range of motion in adults. Orthop. Traumatol. Surg. Res..

[71] Battista R., Pivarnik J., Dummer G., Sauer N., Malina R. (2007). Comparisons of physical characteristics and performances among female collegiate rowers. J. Sports Sci..

[72] Kapandji A. (2019). The Physiology of the Joints: The Upper Limb.

[73] O’Sullivan K., McAuliffe S., DeBurca N. (2012). The effects of eccentric training on lower limb flexibility: A systematic review. Br. J. Sports Med..

[74] Choi S. (2014). Cellular mechanism of eccentric-induced muscle injury and its relationship with sarcomere heterogeneity. J. Exerc. Rehabil..

[75] Fridén J., Lieber R. (2001). Eccentric exercise-induced injuries to contractile and cytoskeletal muscle fibre components. Proceedings of the Acta Physiologica Scandinavica.

[76] Ying J., Cen X., Yu P. (2021). Effects of Eccentric Exercise on Skeletal Muscle Injury: From An Ultrastructure Aspect: A Review. Phys. Act. Health.

[77] Mangine G., Tankersley J., Mcdougle J., Velazquez N., Roberts M., Esmat T., Vandusseldorp T., Feito Y. (2020). Predictors of CrossFit open performance. Sports.

[78] Martínez-Gómez R., Valenzuela P., Alejo L., Gil-Cabrera J., Montalvo-Pérez A., Talavera E., Lucia A., Moral-González S., Barranco-Gil D. (2020). Physiological Predictors of Competition Performance in CrossFit Athletes. Int. J. Environ. Res. Public Health.

[79] Toledo R., Dias M., Toledo R., Erotides R., Pinto D., Reis V., Novaes J., Vianna J., Heinrich K., Schmidt A. (2021). Comparison of Physiological Responses and Training Load between Different CrossFit^®^ Workouts with Equalized Volume in Men and Women. Life.

[80] Keogh J., Hume P., Pearson S. (2006). Retrospective injury epidemiology of one hundred one competitive Oceania power lifters: The effects of age, body mass, competitive standard, and gender. J. Strength Cond. Res..

[81] Sugimoto D., Zwicker R., Quinn B. (2020). Part II: Comparison of CrossFit-related injury presenting to sports medicine clinic by sex and age. Clin. J. Sport Med..

